# Perceptions and use of e-cigarettes among young adults in Hong Kong

**DOI:** 10.1186/s12889-019-7464-z

**Published:** 2019-08-16

**Authors:** Nan Jiang, Charles M. Cleland, Man Ping Wang, Antonio Kwong, Vienna Lai, Tai Hing Lam

**Affiliations:** 1 Department of Population Health, School of Medicine, New York University, 180 Madison Ave, 17th Floor, New York, NY 10016 USA; 2 School of Nursing, The University of Hong Kong, 4/F William MW Mong Block, 21 Sassoon Road, Pok Fu Lam, Hong Kong; 3 Hong Kong Council on Smoking and Health, 4402-03, 44/F, Hopewell Centre, 183 Queen’s Road East, Wan Chai, Hong Kong, Hong Kong; 4 School of Public Health, The University of Hong Kong, G/F, Patrick Manson Building (North Wing), 7 Sassoon Road, Pokfulam, Hong Kong

**Keywords:** Electronic cigarettes, Smoking, Young adult, Susceptibility, Perceptions, Social norms

## Abstract

**Background:**

Little is known about the risk and addiction perceptions of e-cigarettes among Asian populations. We examined e-cigarette perceptions among young adults in Hong Kong and the association between the perceptions and e-cigarette use patterns.

**Methods:**

An online survey was administered to a convenience sample of Hong Kong residents aged 18–35 (*N* = 1186). Measures of e-cigarette perceptions included perceived harm and addictiveness of e-cigarettes, perceived harm of secondhand e-cigarette aerosol, and perceived popularity of e-cigarette use among peers. Separate multinomial logistic regression models were conducted to examine the associations between the four perceptions and former and current use of e-cigarettes relative to never use, controlling for demographics and current cigarette smoking status. Interactions of e-cigarette perceptions and current cigarette smoking were assessed in all models. Among current e-cigarette users, bivariate exact logistic regression models were used to examine the relationships between each of the perceptions and frequent e-cigarette use (≥3 days in past 30-day vs. 1–2 days). Among participants who had never used e-cigarettes, separate multivariable logistic regression models were conducted to examine the associations between e-cigarette perceptions and susceptibility to e-cigarette use.

**Results:**

Overall, 97.2% of participants were aware of e-cigarettes, and 16.1% had tried e-cigarettes (11.3% former users; 4.8% current users). Young adults perceived e-cigarettes (and aerosol) as less harmful, less addictive, and less popular than cigarettes. Current cigarette smokers reported significantly lower perceived harmfulness and addictiveness of e-cigarettes, lower perceived harmfulness of e-cigarette aerosol, and higher perceived popularity than nonsmokers. The lower degree of harm and addiction perceptions, and higher levels of popularity perceptions were associated with greater odds of e-cigarette use, and these relationships were generally stronger among nonsmokers compared to current cigarette smokers. E-cigarette perceptions were not associated with frequent e-cigarette use. Perceiving e-cigarettes (and aerosol) as less harmful and less addictive were associated with greater susceptibility to e-cigarette use. Compared to nonsmokers, current smokers were more likely to report e-cigarette use and susceptibility.

**Conclusions:**

Continued monitoring of e-cigarette use and perceptions is needed. Educational programs should emphasize the potential harmful and addictive properties of e-cigarettes and the risks of secondhand exposure to e-cigarette aerosol.

**Electronic supplementary material:**

The online version of this article (10.1186/s12889-019-7464-z) contains supplementary material, which is available to authorized users.

## Background

E-cigarette use has surged in popularity worldwide, particularly among young people [1, 2]. In the US, for instance, lifetime use of e-cigarettes among adults increased from 1.8% in 2010 to 13.0% in 2013, while current use increased from 0.3 to 6.8% [3]. In 2016, 15.3% of US adults had ever used e-cigarettes, and 3.2% reported current use [4]. Young adults aged 18–24 years had the highest prevalence of e-cigarette use across all age groups [4]. Compared to western countries, e-cigarette use is less prevalent in Asia. In 2015, 3.1 and 0.5% of Chinese residents (aged ≥15 years) living in mainland China reported ever and current use of e-cigarettes respectively [5], 0.7 and 0.2% of Hong Kong residents were ever and current e-cigarette users [6], and 2.7% of Taiwanese had tried e-cigarettes [7]. As in western countries, e-cigarette use in Asian countries concentrates in cigarette smokers and young people [6–8]. The rapid growth of e-cigarette use is largely related to favorable perceptions about e-cigarettes (e.g., reduced harm, lower addictiveness, and higher social acceptability) [9, 10]. As suggested by health behavior theories (e.g., Health Belief Model), perceptions play an important role in influencing people’s behaviors [11].

It is well established that the low degree of perceived harm of e-cigarettes is associated with e-cigarette use. According to the Population Assessment of Tobacco and Health (PATH), the top reasons for e-cigarette use among US adults center around perceptions that e-cigarettes are less harmful than cigarettes to users and other people [12]. In Malaysia, the perception of e-cigarettes as less toxic is the most common reason for e-cigarette use among adults [13]. Longitudinal data [14, 15] and cross-sectional studies [10, 16, 17] have demonstrated that the low harm perceptions of e-cigarettes are associated with increased initiation of e-cigarette use.

The low addiction perception of e-cigarettes and its relationship with e-cigarette use have also been documented in the literature. In a nationally representative sample of US adults, Wiseman et al. [18] found that 48% of e-cigarette users perceived e-cigarettes as less addictive than cigarettes, and the low addiction perception was associated with ever e-cigarette use. In a study among college students, Cooper et al. [19] found that 71% of respondents perceived e-cigarettes as not or somewhat addictive, and the perception was associated with current e-cigarette use.

Research on social norms around e-cigarette use is nascent and findings have been mixed. Measures of social norms have focused on either descriptive norms (perceived prevalence of e-cigarette use) or injunctive norms (perceived social acceptability of e-cigarettes) [20]. Texan adolescents generally perceive e-cigarettes as more popular and more acceptable than cigarettes among their peers and close friends [21]. Whereas adolescents in Mexico perceive e-cigarettes as less popular than cigarettes among friends and family [22]. College students in Southeastern US perceive e-cigarettes as less acceptable than cigarettes [23]. Regarding the link between social norms and e-cigarette use, Yang et al. [24] reported that the higher perceived prevalence of e-cigarette use was associated with current e-cigarette use among US young people (aged 13–25). Whereas Waters et al. [10] found that, among US adults, higher perceived acceptability of e-cigarettes was associated with current e-cigarette use in the unadjusted regression model only.

Understanding young adults’ perceptions of e-cigarettes can help understand the mechanisms through which young people become interested in e-cigarettes. It can help inform regulatory policies on e-cigarettes and educational interventions that address e-cigarette use. In Hong Kong, e-cigarettes entered the marketplace around 2008. Awareness of e-cigarettes is high, with 84% of residents reporting having heard of e-cigarettes in 2015 [6]. However, the prevalence of e-cigarette use is low in Hong Kong. Nicotine-containing e-cigarettes are classified as a pharmaceutical product that is prohibited to sell without approval from the government. As of July 2019, there are no nicotine-containing e-cigarettes approved for sale and distribution in Hong Kong. However, nicotine-free e-cigarettes are widely available in retail stores and through online platforms (e.g., social media and online stores). People can easily purchase nicotine-containing e-cigarettes through the Internet. Legislation to ban the import, manufacture, sale, distribution, and promotion of e-cigarettes is currently under consideration in Hong Kong.

Published data on perceptions of e-cigarettes among Asian populations is limited. How young adults in Asian countries think about e-cigarettes, and how their perceptions affect e-cigarette use behaviors remain unknown. Furthermore, there is a paucity of research on the link between e-cigarette perceptions and the frequency and susceptibility of e-cigarette use. This study examined the perceptions of e-cigarettes (including perceived harm, addictiveness, and social norms) among young adults in Hong Kong, and the association between these perceptions and e-cigarette use behaviors (including use status, frequency, and susceptibility).

## Methods

### Participants and procedure

We collected data through an online survey (Additional file 1) in two stages. We oversampled current cigarette smokers to compare e-cigarette perceptions and use patterns by cigarette smoking status. Data were collected by the University of Hong Kong’s Public Opinion Programme (POP), an academic agency specialized in collecting data on public opinions.

In Stage 1 (November 2016), POP sent an invitation email to a panel of 5237 Hong Kong residents who were recruited for POP’s previous and other ongoing research projects. The invitation email provided information on study purpose and eligibility criteria (i.e., Hong Kong residents aged 18–35), and included a link to an online consent form. People who confirmed their eligibility and completed the online consent form were directed to a 22-item online survey that was developed for this study. Participants could choose survey language based on their preference (i.e., English, or traditional or simplified Chinese). As an incentive for participation, the first 200 respondents who submitted a completed survey received a HK$50 gift card (≈US$6.4, exchange rate: US$1 = HK$7.8 in 2016/17). A total of 1049 surveys were received, yielding a crude response rate of 20% in Stage 1.

In Stage 2 (December 2016–March 2017), POP added one eligibility criterion (i.e., past 30-day cigarette smokers) and sent the invitation email to another 37,050 Hong Kong residents, and 315 current cigarette smokers completed the survey, yielding a crude response rate of 0.9% in Stage 2. We were not able to determine the number of young adult current smokers, the true denominator for the response rate. Therefore the adjusted response rate was unknown. As an incentive, the first 50 respondents who completed the survey in Stage 2 received a HK$50 gift card.

In total, 1364 surveys were received. We excluded 178 surveys due to incomplete submissions (with more than 50% missing items) and missing cigarette and e-cigarette use questions. Thus, 1186 responses were included in the analysis. The study was approved by the Institutional Review Board of the University of Hong Kong/Hospital Authority Hong Kong West Cluster (UW 16–349).

### Measures

Demographics included age, gender, education, and place of birth. Awareness of e-cigarettes was assessed by asking, “Have you ever heard of electronic cigarettes prior to the survey?” with a brief description of e-cigarettes and a picture showing different types of e-cigarettes (yes/no).

E-cigarette use was derived from two items assessing ever use (“Have you ever used an electronic cigarette, even just one time?” with answer options of “Yes”, “No”, and “Refuse to answer”) and current use (“During the past 30 days, on how many days did you use an electronic cigarette?” with a blank space for numeric response). Participants were categorized as “never users”, “former users” (ever use but no current use), and “current users” (ever and current use). Following the previous research on e-cigarettes among youth [25, 26], current e-cigarette users were dichotomized as “infrequent users” (1–2 days) or “frequent users” (≥3 days). There is no standard definition for frequent e-cigarette use. We defined frequent use as “≥3 days” to differentiate experimenters from those who were less likely to be experimenters. Current cigarette smoking was assessed by asking, “During the past 30 days, did you smoke cigarettes, even for one puff?” (yes/no).

Measures on e-cigarette perceptions were adapted from PATH surveys. Three items assessed harm and addiction perceptions (“How harmful do you think each of the following tobacco products is to the health of the smoker?”, “How harmful do you think the secondhand smoke or the vapor is to other people for each of the following tobacco products?”, and “How addictive do you think each of the following tobacco products is?”). One item assessed the descriptive norms by asking “How popular among your peers do you think each of the following tobacco products is?” For each item, participants were asked to rate on a 0–10 visual analog scale for e-cigarette, cigarette, and waterpipe, respectively (“0” labeled as “not harmful/addictive/popular at all”, and “10” as “extremely harmful/addictive/popular”). Participants could choose “Don’t know” as an answer option for each item.

Susceptibility to e-cigarette use was measured by two items (“Do you think you will try an electronic cigarette in the next 12 months?”, “If one of your friends was to offer you an electronic cigarette, would you use it?”) with response options of “definitely yes”, “probably yes”, “probably no”, “definitely no”, and “Don’t know”. Participants who answered “definitely no” to both items were considered unsusceptible to e-cigarette use, and those who answered otherwise were considered susceptible.

### Statistical analyses

Stata version 14.2 was used for data analyses. Sample characteristics were summarized using descriptive statistics. Fisher’s exact tests (for categorical variables) and t-tests (for continuous variables) were conducted to compare e-cigarette awareness, perceptions, and use patterns by current cigarette smoking status. Among respondents who were aware of e-cigarettes, paired sample t-tests were used to compare perceptions of e-cigarettes and cigarettes. Separate multinomial logistic regression models were conducted to examine whether the four perceptions of e-cigarettes were associated with former and current e-cigarette use (relative to never use), controlling for age, gender, education, place of birth, and current cigarette smoking status. Interactions between e-cigarette perceptions and current cigarette smoking status were tested in all models. When the interaction was significant, we reported estimates by current cigarette smoking status; when the interaction was not significant, we reported the overall estimates only.

Next, subgroup analyses were conducted. Among current e-cigarette users, bivariate exact logistic regression models were used to assess the association between each of the four perceptions and frequent e-cigarette use by current cigarette smoking status. Among e-cigarette never users who were aware of e-cigarettes, separate multivariable logistic regression models were conducted to examine the association between e-cigarette perceptions and susceptibility to e-cigarette use, controlling for demographics and current cigarette smoking. Interactions between e-cigarette perceptions and current cigarette smoking were tested in all of the models.

## Results

The respondents included 811 nonsmokers and 375 current cigarette smokers (Table 1). With an average age of 24.2 years (SD = 5.14), our sample was nearly evenly split by gender (54.7% females). Most had a bachelor’s degree (58.9%) and were born in Hong Kong (75.0%). The majority of respondents (97.2%) were aware of e-cigarettes, and awareness was similar for current cigarette smokers and nonsmokers (*p* = .051). About 16.1% of respondents reported having used e-cigarettes, including 11.3% former users and 4.8% current users. E-cigarette use was more prevalent among current cigarette smokers than nonsmokers (former e-cigarette use: 24.5% vs. 5.2%, *p* < .001; current e-cigarette use: 12.3% vs. 1.4%, *p* < .001). Current e-cigarette use was more prevalent in males than females, although the difference was not significant (6.2% vs. 3.7%, *p* = .052). But no difference was detected in former e-cigarette use by gender (11.4% vs. 11.3%, *p* = .39; data not shown).

**Table 1:** Sample characteristics, perceptions, and use of e-cigarettes among young adults in Hong Kong

	Total sample (N = 1186)		Current cigarette smoking status^a^				*p*
			Nonsmoker (*n* = 811)		Current smoker (*n* = 375)		
	n	(%)	n	(%)	n	(%)	
Age, Mean (SD)	24.18	(5.14)	24.41	(5.29)	23.68	(4.78)	.024
Gender							.005
Male	537	(45.3)	345	(42.5)	192	(51.2)	
Female	649	(54.7)	466	(57.5)	183	(48.8)	
Education attainment							.074
High school or less	145	(12.2)	96	(11.8)	49	(13.1)	
Some college, no degree	126	(10.6)	94	(11.6)	32	(8.5)	
Bachelor’s degree	699	(58.9)	488	(60.2)	211	(56.3)	
Advanced degree	205	(17.3)	125	(15.4)	80	(21.3)	
Unreported	11	(0.9)	8	(1.0)	3	(0.8)	
Place of birth							<.001
Hong Kong	889	(75.0)	640	(78.9)	249	(66.4)	
Mainland China	186	(15.7)	119	(14.7)	67	(17.9)	
Others	94	(7.9)	43	(5.3)	51	(13.6)	
Unreported	17	(1.4)	9	(1.1)	8	(2.1)	
Awareness of e-cigarettes							.051
Yes	1153	(97.2)	782	(96.4)	371	(98.9)	
No	25	(2.1)	22	(2.7)	3	(0.8)	
Don’t know/not sure	8	(0.7)	7	(0.9)	1	(0.3)	
E-cigarette use^b^							<.001
Never user	983	(82.9)	755	(93.1)	228	(60.8)	
Former user	134	(11.3)	42	(5.2)	92	(24.5)	
Current user	57	(4.8)	11	(1.4)	46	(12.3)	
Undetermined	12	(1.0)	3	(0.4)	9	(2.4)	
Frequency of e-cigarette use^c^							.971
1–2 days in past 30 days	36	(63.2)	7	(63.6)	29	(63.0)	
≥ 3 days in past 30 days	21	(36.8)	4	(36.4)	17	(37.0)	
Susceptibility to e-cigarette use^d^							<.001
Yes	323	(34.0)	155	(21.4)	168	(75.0)	
No	617	(65.0)	563	(77.6)	54	(24.1)	
Unreported	10	(1.1)	8	(1.1)	2	(0.9)	
E-cigarette perceptions^e^, mean (SD)							
Perceived harm of e-cigarettes	7.59	(2.34)	8.07	(2.13)	6.58	(2.44)	<.001
Perceived harm of e-cigarette aerosol	6.93	(2.93)	7.62	(2.67)	5.55	(2.94)	<.001
Perceived addictiveness of e-cigarettes	7.22	(2.70)	7.91	(2.38)	5.77	(2.78)	<.001
Perceived popularity of e-cigarette use	2.62	(2.87)	2.34	(2.91)	3.23	(2.74)	<.001

*Notes.* Age and e-cigarette perceptions are reported as mean and SD in parenthesis. Other variables are reported as n and % in parenthesis

^a^Current smokers reported smoking cigarettes on at least 1 day in the past 30 days; nonsmokers reported smoking 0 days in the past 30 days

^b^E-cigarette never users reported having never used e-cigarettes; former users reported having ever used e-cigarettes but not in the past 30 days; current users reported using e-cigarettes on at least 1 day in the past 30 days

^c^Among current e-cigarettes users (n = 57)

^d^Among respondents who were aware of e-cigarettes but had never used e-cigarettes (*n* = 950)

^e^Among respondents who were aware of e-cigarettes (*n* = 1153)

Among current e-cigarette users, 36.8% reported frequent e-cigarette use. There was no difference in frequent e-cigarette use between current cigarette smokers and nonsmokers (36.4% vs. 37.0%, *p* = .97). Among e-cigarette never users, 34.0% were susceptible to e-cigarette use, and the susceptibility among current cigarette smokers was 3.5-fold that for nonsmokers (75.0% vs. 21.4%, *p* < .001).

E-cigarettes were perceived as less harmful than cigarettes with 4.1% reporting “Don’t know” (7.59, SD = 2.34 vs. 9.05, SD = 1.46; *p* < .001; data on cigarette perceptions are not shown); less addictive with 7.4% “Don’t know” (7.22, SD = 2.70 vs. 8.57, SD = 1.94; *p* < .001); and less popular with 5.3% “Don’t know” (2.62, SD = 2.87 vs. 4.97, SD = 3.40; *p* < .001). E-cigarette aerosol was perceived as less harmful than secondhand cigarette smoke with 7.7% reporting “Don’t know” (6.93, SD = 2.93 vs. 9.11, SD = 1.46; *p* < .001). Compared to nonsmokers, current cigarette smokers reported significantly lower perceived harm of e-cigarettes and aerosol, lower perceived addictiveness of e-cigarettes, and higher perceived popularity of e-cigarette use among their peers (*p* < .001).

Respondents who perceived more harm from e-cigarettes had decreased odds of both former and current use of e-cigarettes, relative to never use (Table 2), and this relationship was stronger in nonsmokers (former e-cigarette use: adjusted odds ratio [AOR] = 0.70, 95% confidence interval [CI]: 0.61–0.81, *p* < .001; current e-cigarette use: AOR = 0.63, 95% CI: 0.49–0.81, *p* < .001) than current cigarette smokers (former e-cigarette use: AOR = 0.90, 95% CI: 0.80–1.00, *p* = .055; current e-cigarette use: AOR = 0.80, 95% CI: 0.69–0.92, *p* = .002). Similarly, respondents who perceived more harm from e-cigarette aerosol and greater addictiveness of e-cigarettes had decreased odds of e-cigarette use, and these relationships were stronger among nonsmokers than current cigarette smokers. Respondents who perceived higher popularity of e-cigarettes had increased odds of e-cigarette use, and the relationship was similar for current cigarette smokers and nonsmokers. In all of the models with combined smokers and nonsmokers, current cigarette smokers had increased odds of former and current e-cigarette use than nonsmokers (*p* < .001 in all models). Gender was not related to e-cigarettes use (*p* = .14–.74).

**Table 2:** Perceptions and use of e-cigarettes among young adults

	Former e-cigarette use^a^				Current e-cigarette use^b^			
	AOR^c^ [95% CI]	*p*	AOR^c^ [95% CI]	*p*	AOR^c^ [95% CI]	*p*	AOR^c^ [95% CI]	*p*
	Among nonsmokers^d^		Among current cigarette smokers^e^		Among nonsmokers^d^		Among current cigarette smokers^e^	
Perceived harm of e-cigarettes (*n* = 734 for nonsmokers and 338 for current cigarette smokers)	0.70 [0.61, 0.81]	<.001	0.90 [0.80, 1.00]	.055	0.63 [0.49, 0.81]	<.001	0.80 [0.69, 0.92]	.002
Perceived harm of e-cigarette aerosol (*n* = 696 for nonsmokers and 334 for current cigarette smokers)	0.73 [0.65, 0.82]	<.001	0.89 [0.81, 0.98]	.017	0.58 [0.45, 0.75]	<.001	0.88 [0.78, 0.99]	.041
Perceived addictiveness of e-cigarettes (*n* = 707 for nonsmokers and 328 for current cigarette smokers)	0.69 [0.62, 0.78]	<.001	0.93 [0.84, 1.02]	.119	0.63 [0.51, 0.79]	<.001	0.84 [0.75, 0.95]	.005
	Former e-cigarette use^a^				Current e-cigarette use^b^			
	AOR^f^ [95% CI]		*p*		AOR^f^ [95% CI]		*p*	
Perceived popularity of e-cigarette use (*n* = 1059)	1.09 [1.02, 1.17]		.013		1.22 [1.11, 1.34]		<.001	

*Notes.* AOR = adjusted odds ratio; CI = confidence interval

^a^E-cigarette former users reported having ever used e-cigarettes but not in the past 30 days

^b^E-cigarette current users reported using e-cigarettes on at least 1 day in the past 30 days

^c^AORs for former and current e-cigarette use in reference to never use; multinomial logistic regression models controlled for age, gender, education attainment, and place of birth

^d^Nonsmokers reported smoking cigarettes on 0 days in the past 30 days

^e^Current cigarette smokers reported smoking cigarettes on at least 1 day in the past 30 days

^f^AORs for former and current e-cigarette use in reference to never use; multinomial logistic regression models controlled for age, gender, education attainment, place of birth, and current cigarette smoking status

In general, e-cigarette perceptions were not related to frequent e-cigarette use (Table 3). But among current cigarette smokers, those who perceived e-cigarettes as more addictive had decreased odds of reporting frequent e-cigarette use compared to those who perceived e-cigarettes as less addictive (odds ratio[OR] = 0.77, 95% CI: 0.59–0.97, *p* = .03). Current cigarette smoking status was unrelated to frequent e-cigarette use (OR = 1.03, 95% CI: 0.22–5.50, *p* = 1.00; data not shown).

**Table 3:** Perceptions of e-cigarettes and frequency of e-cigarette use among current e-cigarette users

	Frequent e-cigarette use^a^					
	OR	[95% CI]	*p*	OR	[95% CI]	*p*
	Among nonsmokers^b^			Among current cigarette smokers^c^		
Perceived harm of e-cigarettes (n = 11 for nonsmokers and 45 for current cigarette smokers)	0.54	[0.93, 1.46]	.333	0.87	[0.65, 1.13]	.300
Perceived harm of e-cigarette aerosol (*n* = 10 for nonsmokers and 44 for current cigarette smokers)	0.77	[0.35, 1.36]	.476	0.82	[0.62, 1.04]	.099
Perceived addictiveness of e-cigarettes (n = 11 for nonsmokers and 46 for current cigarette smokers)	1.11	[0.68, 1.89]	.758	0.77	[0.59, 0.97]	.026
Perceived popularity of e-cigarette use (n = 11 for nonsmokers and 46 for current cigarette smokers)	1.24	[0.79, 2.07]	.382	1.03	[0.82, 1.30]	.819

*Notes.* OR = odds ratio; CI = confidence interval

^a^Frequent e-cigarette use was defined as e-cigarette use on 3 or more days in the past 30 days

^b^Nonsmokers reported smoking cigarettes on 0 days in the past 30 days

^c^Current cigarette smokers reported smoking cigarettes on at least 1 day in the past 30 days

Among e-cigarette never users, those who perceived more harm from e-cigarettes and aerosol and greater addictiveness of e-cigarettes had lower odds of e-cigarette susceptibility (Table 4), and these relationships were similar for current cigarette smokers and nonsmokers. The perceived popularity of e-cigarettes was unrelated to e-cigarette susceptibility. Current cigarette smokers had increased odds of reporting e-cigarette susceptibility compared to nonsmokers (*p* < .001 in all models).

**Table 4:** Perceptions of e-cigarettes and susceptibility to use among never users

	Susceptibility to e-cigarette use		
	AOR^a^	[95% CI]	*p*
Perceived harm of e-cigarettes (*n* = 887)	0.73	[0.67, 0.79]	<.001
Perceived harm of e-cigarette aerosol (*n* = 848)	0.80	[0.75, 0.85]	<.001
Perceived addictiveness of e-cigarettes (*n* = 847)	0.78	[0.73, 0.84]	<.001
Perceived popularity of e-cigarette use (*n* = 873)	0.99	[0.93, 1.05]	.723

*Notes.* AOR = adjusted odds ratio; CI = confidence interval

^a^Multivariable logistic regression model controlled for age, gender, education attainment, place of birth, and current cigarette smoking status

## Discussion

This is the first study to comprehensively assess e-cigarette perceptions in a sample of Asian young adults. The majority (97.2%) of young adults were aware of e-cigarettes. E-cigarette use was more prevalent in our sample than the general adult population in Hong Kong. It might be that we oversampled current cigarette smokers and our sample was composed of young adults only.

Young adults, particularly current cigarette smokers, perceived e-cigarettes as less harmful and less addictive than cigarettes, and perceived e-cigarette aerosol as less hazardous than secondhand cigarette smoke. Our findings are consistent with earlier studies on e-cigarette perceptions among young adults [23, 27, 28]. Educational programs should inform people that, although e-cigarettes typically contain fewer chemicals than conventional cigarettes, e-cigarettes and aerosol are not harmless [29]. E-cigarettes contain and emit numerous potentially toxic substances (e.g., heavy metals and volatile organic compounds) in addition to nicotine which has been detected in most e-cigarettes including those labeled as nicotine-free [29].

Both cigarette smokers and nonsmokers perceived e-cigarettes as less popular among peers than cigarettes. Compared to nonsmokers, current cigarette smokers perceived e-cigarettes as more popular. Findings reflect the tobacco landscape in Hong Kong, where e-cigarettes are less common than cigarettes. In 2015, 0.2% of Hong Kong residents reported current e-cigarette use [6], whereas 10.5% reported current daily cigarette smoking [30]. Despite the low prevalence, we recommend continued monitoring of e-cigarette use and public perceptions, particularly among young people, as data will provide evidence for developing health messages and inform e-cigarette regulations.

Consistent with previous studies among young adults [16, 19, 31], we found that the lower harm and addiction perceptions of e-cigarettes and aerosol, and the higher popularity perceptions were associated with greater odds of e-cigarette use. Educational campaigns need to inform young people about the potential harmful and addictive properties of e-cigarettes, and the risks of secondhand exposure to e-cigarette aerosol. Messages emphasizing the uncertainty of long-term health consequences of e-cigarette use may discourage young adults from using e-cigarettes. We do not know why the relationships between harm and addiction perceptions and e-cigarette use are stronger in nonsmokers compared to current cigarette smokers. Longitudinal research is needed to examine the role of e-cigarette perceptions on the initiation and intensity of e-cigarette use, and how the relationship differs by cigarette smoking status.

Interestingly, most current e-cigarette users were infrequent users, and e-cigarette perceptions were generally unrelated to frequent e-cigarette use. Our finding contrasts with a prior longitudinal study of US young adults which found that the low harm perception of e-cigarettes predicted more frequent e-cigarette use [32]. It is possible that young adult e-cigarette users in our study are primarily experimenters or casual users who have no goal-oriented reasons (e.g., curiosity) as the most common reasons for e-cigarette use among Hong Kong adults are seeing friends using it or being attracted by e-cigarettes [7], whereas e-cigarette users in Brikmanis et al’s study may be primarily motivated users who have goal-oriented reasons (e.g., to circumvent smokefree laws). Motivated users and casual users have different patterns of e-cigarette use. For instance, among US adults, casual users are more likely to stop using e-cigarettes than motivated users [33]. Among Korean adolescents, e-cigarette use is less frequent among casual users than motivated users [34]. Future researchers may ask the reasons for e-cigarette use to have a thorough understanding of e-cigarette use behavior.

One-third (34%) of never users of e-cigarettes were susceptible to using e-cigarettes. It is of concern as a longitudinal study has found that e-cigarette susceptibility predicts the initiation of e-cigarette use among US adolescents [35]. Future research should affirm whether e-cigarette susceptibility leads to subsequent e-cigarette use among Asian populations. Consistent with prior research, our study confirmed that the low harm and addiction perceptions of e-cigarettes and aerosol were associated with increased odds of e-cigarette susceptibility. Prior studies revealed that US youth and young adults who perceived e-cigarettes as less harmful and less addictive were more likely to be susceptible to e-cigarette use than those who believe otherwise [36, 37]. Tung et al. [38] found that Hong Kong adolescents perceiving e-cigarettes as less harmful to users and others were more likely to report e-cigarette susceptibility. These findings collectively suggest that, to prevent the initiation of e-cigarette use, interventions must address young people’s perceptions by educating them about the harmful and addictive nature of e-cigarettes.

The perceived popularity of e-cigarette use was unrelated to e-cigarette susceptibility. Our finding is inconsistent with a study of Mexican adolescents which found that perceived higher popularity of e-cigarette use was associated with greater susceptibility to e-cigarette use [22]. The different findings may be explained by the difference in sample populations and measures of descriptive norms. In the Mexican study, descriptive norm was a dichotomized measure that indicated whether adolescents’ family and best friends used e-cigarettes. In our study, descriptive norm was a number ranging from 0 to 10 that estimated the perceived popularity of e-cigarettes among young adults’ peers. Give the novelty of e-cigarettes, formative research may help develop measures to more accurately assess social norms around e-cigarette use.

Not surprisingly, current cigarette smokers were more likely to report e-cigarette use and susceptibility than nonsmokers, which is consistent with the literature [9, 16, 31, 37]. We observed no relationship between cigarette smoking and frequent e-cigarette use. It is possible that our statistical power is not sufficient due to the small number of current e-cigarette users (*n* = 57). Future research with larger sample sizes of e-cigarette users would be desirable to examine the effect of cigarette smoking on e-cigarette use trajectories among Asian populations.

Gender was found to be unrelated to e-cigarette use. It contrasts with our previous study conducted in a representative sample of Hong Kong adults which concluded that ever e-cigarette use was more prevalent among males [8]. The inconsistent findings suggest that correlates of e-cigarette use may be different among young versus middle-aged and older adults. Future research is warranted to better characterize e-cigarette use behaviors in different subgroups.

This study has certain limitations. First, owing to the cross-sectional study nature, causal inferences cannot be drawn regarding the association between e-cigarette perceptions and use behaviors. Second, due to the convenience sample of young adults who were recruited online, findings may not be generalizable to the entire young adult population in Hong Kong. Third, the low crude response rates, particularly among current cigarette smokers, might suggest response bias. However, we could not ascertain the true number of eligible people and therefore, we could not determine the adjusted response rates. Fourth, the small size of current e-cigarette users made our estimate of this subgroup unstable. Lastly, self-reports were subject to recall and reporting errors.

## Conclusions

This study contributes important knowledge on perceptions of e-cigarettes in a sample of Asian young adults. Findings suggest that strategic interventions need to communicate with young adults about the harmful and addictive properties of e-cigarettes and the adverse health consequences of secondhand exposure to e-cigarette aerosol. Messages emphasizing the uncertainty of long-term effects of e-cigarette use may deter young people from using e-cigarettes.

## Additional file

Additional file 1: Survey on the Perceptions and Use of Electronic Cigarettes and Waterpipe among Young Adults in Hong Kong. Survey of the study (English version). (DOCX 42 kb)

## Data Availability

The datasets used and/or analyzed during the current study are available from the corresponding author on reasonable request.

## Abbreviations

AOR: adjusted odds ratio
CI: confidence interval
OR: odds ratio
PATH: Population Assessment of Tobacco and Health
POP: Public Opinion Programme
