# Supplementary material for: Perceptions and use of e-cigarettes among young adults in Hong Kong
Source: BMC Public Health. 2019 Aug 16;19:1123. doi: 10.1186/s12889-019-7464-z (PMC6697992; doi:10.1186/s12889-019-7464-z)
Supplement: Supplementary file 1 — Survey on the Perceptions and Use of Electronic Cigarettes and Waterpipe among Young Adults in Hong Kong. Survey of the study (English version). (DOCX 42 kb) [file 12889_2019_7464_MOESM1_ESM.docx]

**Survey on the Perceptions and Use of Electronic Cigarettes and**

**Waterpipe among Young Adults in Hong Kong**

School of Public Health (SPH) at The University of Hong Kong commissioned Public Opinion Programme (POP) at The University of Hong Kong to conduct this online “Survey on the Perceptions and Use of Electronic Cigarettes and Waterpipe / Shisha / Hookah”. The target population of this survey is Hong Kong residents aged 18-35. The survey aimed to understand Hong Kong young adults’ awareness, use, and perceptions about electronic cigarettes and waterpipe / shisha / hookah. All information you provided will be kept strictly confidential and used for aggregate analysis only. For any enquiry regarding the survey, please feel free to contact Ms. Chan at 3917 7725. If you want to know more about the rights as a participant, please contact the Institutional Review Board of the University of Hong Kong/Hospital Authority Hong Kong West Cluster (HKU/HA HKW IRB) at 2255 4086 during office hours (IRB Reference Number: UW 16-349).

It will take 10 minutes or less to complete this questionnaire. If you are willing to participate in our study, please complete the questionnaire. The first 200 participants who completed and submitted the questionnaire will receive a HK$50 Starbucks e-gift card as a token of appreciation. All your personal information will be kept confidential, and be destroyed within 6 months after the survey is complete. Your participation is totally voluntary.

S1 I understand the above information and agree to participate in this research. □ Yes

S2 I am a Hong Kong resident of age 18-35. □ Yes

**Survey on the Perceptions and Use of Electronic Cigarettes and**

**Waterpipe among Young Adults in Hong Kong**

1. **Personal information**
2. Age: ______ years old
3. Gender:

⃝ 1. Male

⃝ 2. Female

1. Education:

⃝ 1. Primary school or lower

⃝ 2. Middle school

⃝ 3. High school, no diploma

⃝ 4. High school graduate

⃝ 5. Secondary 7 (Matriculation)

⃝ 6. Some college with no degree

⃝ 7. Bachelor’s degree

⃝ 8. Postgraduate degree

⃝ 99. Refuse to answer

1. Place of birth:

⃝ 1. Hong Kong

⃝ 2. Mainland China

⃝ 3. Macau

⃝ 4. Taiwan

⃝ 5. Others (Please specify ______________)

⃝ 99. Refuse to answer

1. How long have you lived in Hong Kong?

⃝ 1. Less than 6 months

⃝ 2. 6 months or more

⃝ 99. Refuse to answer

1. During the past 30 days, did you smoke cigarettes, even for one puff?

⃝ 1. Yes

⃝ 2. No

1. **Awareness, Use and Perceptions about Tobacco Products**
2. Have you ever heard of electronic cigarettes prior to this survey? (Electronic cigarettes are an electronic device, by heating of a solution (usually contains nicotine, propylene glycol, glycerin and odorants) for user to inhale.)

⃝ 1. Yes

⃝ 2. No

⃝ 99. Don’t know / not sure

1. Have you ever heard of waterpipe (also called shisha or hookah) prior to this survey? (Waterpipe is a device used to smoke charcoal-heated tobacco where smoke is passed through water prior to inhalation.)

⃝ 1. Yes

⃝ 2. No

⃝ 99. Don’t know / not sure

1. Have you ever used any of the following tobacco products, even just one time?

|  | Yes | No | Refuse to answer |
| --- | --- | --- | --- |
| Traditional cigarette | ⃝ | ⃝ | ⃝ |
| Electronic cigarette | ⃝ | ⃝ | ⃝ |
| Waterpipe / shisha / hookah | ⃝ | ⃝ | ⃝ |
| Cigar | ⃝ | ⃝ | ⃝ |
| Others (Please specify _________ ) | ⃝ | ⃝ | ⃝ |

1. During the past **30 DAYS**, on how many days did you use the following tobacco products?

|  |  | None | Refuse to answer |
| --- | --- | --- | --- |
| Traditional cigarette | _____ days | ⃝ | ⃝ |
| Electronic cigarette | _____ days | ⃝ | ⃝ |
| Waterpipe / shisha / hookah | _____ days | ⃝ | ⃝ |
| Cigar | _____ days | ⃝ | ⃝ |
| Others (Please specify _________ ) | _____ days | ⃝ | ⃝ |

1. Do you think you will try an electronic cigarette or waterpipe in the next 12 months?

|  | Definitely Yes | Probably Yes | Probably No | Definitely No | I have never heard about this tobacco product | Refuse to answer |
| --- | --- | --- | --- | --- | --- | --- |
| Electronic cigarette | ⃝ | ⃝ | ⃝ | ⃝ | ⃝ | ⃝ |
| Waterpipe / shisha / hookah | ⃝ | ⃝ | ⃝ | ⃝ | ⃝ | ⃝ |

1. If one of your friends was to offer you an electronic cigarette or invite you to use waterpipe, would you use it?

|  | Definitely Yes | Probably Yes | Probably No | Definitely No | I have never heard about this tobacco product | Refuse to answer |
| --- | --- | --- | --- | --- | --- | --- |
| Electronic cigarette | ⃝ | ⃝ | ⃝ | ⃝ | ⃝ | ⃝ |
| Waterpipe / shisha / hookah | ⃝ | ⃝ | ⃝ | ⃝ | ⃝ | ⃝ |

1. How harmful do you think each of the following tobacco products is to the health of the smoker? Please rate the harm of each tobacco products on a 0-10 scale, where 0 means not harmful at all, 10 means extremely harmful, and 5 means half-half.

|  | Not harmful  at all  0 | 1 | 2 | 3 | 4 | 5 | 6 | 7 | 8 | 9 | Extremely harmful  10 | I have never heard about this tobacco product | Don’t know |
| --- | --- | --- | --- | --- | --- | --- | --- | --- | --- | --- | --- | --- | --- |
| Traditional cigarette | ⃝ | ⃝ | ⃝ | ⃝ | ⃝ | ⃝ | ⃝ | ⃝ | ⃝ | ⃝ | ⃝ | ⃝ | ⃝ |
| Electronic cigarette | ⃝ | ⃝ | ⃝ | ⃝ | ⃝ | ⃝ | ⃝ | ⃝ | ⃝ | ⃝ | ⃝ | ⃝ | ⃝ |
| Waterpipe / shisha / hookah | ⃝ | ⃝ | ⃝ | ⃝ | ⃝ | ⃝ | ⃝ | ⃝ | ⃝ | ⃝ | ⃝ | ⃝ | ⃝ |

1. How harmful do you think the secondhand smoke or the vapor are to other people surrounding the smoker for each of the following tobacco products? Please rate on a 0-10 scale, where 0 means not harmful at all, 10 means extremely harmful, and 5 means half-half.

|  | Not harmful  at all  0 | 1 | 2 | 3 | 4 | 5 | 6 | 7 | 8 | 9 | Extremely harmful  10 | I have never heard about this tobacco product | Don’t know |
| --- | --- | --- | --- | --- | --- | --- | --- | --- | --- | --- | --- | --- | --- |
| Traditional cigarette | ⃝ | ⃝ | ⃝ | ⃝ | ⃝ | ⃝ | ⃝ | ⃝ | ⃝ | ⃝ | ⃝ | ⃝ | ⃝ |
| Electronic cigarette | ⃝ | ⃝ | ⃝ | ⃝ | ⃝ | ⃝ | ⃝ | ⃝ | ⃝ | ⃝ | ⃝ | ⃝ | ⃝ |
| Waterpipe / shisha / hookah | ⃝ | ⃝ | ⃝ | ⃝ | ⃝ | ⃝ | ⃝ | ⃝ | ⃝ | ⃝ | ⃝ | ⃝ | ⃝ |

1. How addictive do you think each of the following tobacco products is? Please rate on a scale of 0 to 10, where 0 means not addictive at all, 10 means extremely addictive, and 5 means half-half.

|  | Not addictive  at all  0 | 1 | 2 | 3 | 4 | 5 | 6 | 7 | 8 | 9 | Extremely addictive  10 | I have never heard about this tobacco product | Don’t know |
| --- | --- | --- | --- | --- | --- | --- | --- | --- | --- | --- | --- | --- | --- |
| Traditional cigarette | ⃝ | ⃝ | ⃝ | ⃝ | ⃝ | ⃝ | ⃝ | ⃝ | ⃝ | ⃝ | ⃝ | ⃝ | ⃝ |
| Electronic cigarette | ⃝ | ⃝ | ⃝ | ⃝ | ⃝ | ⃝ | ⃝ | ⃝ | ⃝ | ⃝ | ⃝ | ⃝ | ⃝ |
| Waterpipe / shisha / hookah | ⃝ | ⃝ | ⃝ | ⃝ | ⃝ | ⃝ | ⃝ | ⃝ | ⃝ | ⃝ | ⃝ | ⃝ | ⃝ |

1. How popular among your peers do you think each of the following tobacco products is? Please rate on a 0-10 scale, where 0 means not popular at all, 10 means extremely popular, and 5 means half-half

|  | Not popular  at all  0 | 1 | 2 | 3 | 4 | 5 | 6 | 7 | 8 | 9 | Extremely popular  10 | I have never heard about this tobacco product | Don’t know |
| --- | --- | --- | --- | --- | --- | --- | --- | --- | --- | --- | --- | --- | --- |
| Traditional cigarette | ⃝ | ⃝ | ⃝ | ⃝ | ⃝ | ⃝ | ⃝ | ⃝ | ⃝ | ⃝ | ⃝ | ⃝ | ⃝ |
| Electronic cigarette | ⃝ | ⃝ | ⃝ | ⃝ | ⃝ | ⃝ | ⃝ | ⃝ | ⃝ | ⃝ | ⃝ | ⃝ | ⃝ |
| Waterpipe / shisha / hookah | ⃝ | ⃝ | ⃝ | ⃝ | ⃝ | ⃝ | ⃝ | ⃝ | ⃝ | ⃝ | ⃝ | ⃝ | ⃝ |

1. Compared to smoking traditional cigarettes, I think that electronic cigarette use is ____.

⃝ 1. Much less harmful

⃝ 2. Less harmful

⃝ 3. As harmful as traditional cigarettes

⃝ 4. More harmful

⃝ 5. Much more harmful

⃝ 8886. I have never heard about electronic cigarette

⃝ 8888. Don’t know

1. Compared to smoking traditional cigarettes, I think that waterpipe / shisha / hookah use is ____.

⃝ 1. Much less harmful

⃝ 2. Less harmful

⃝ 3. As harmful as traditional cigarettes

⃝ 4. More harmful

⃝ 5. Much more harmful

⃝ 8886. I have never heard about waterpipe

⃝ 8888. Don’t know

1. **Marketing of Tobacco Products**
2. During the past 6 months, did you see advertising or promotions for electronic cigarettes in the following channels in Hong Kong?

|  | Never | 1  time | 2  times | 3  times | 4 or more times | Don’t remember / don’t know |
| --- | --- | --- | --- | --- | --- | --- |
| Convenience store (e.g., 7-11, Circle K, Vingo, etc.), local store, market, supermarket or gas station | ⃝ | ⃝ | ⃝ | ⃝ | ⃝ | ⃝ |
| Internet (e.g., taobao, YouTube, etc.) | ⃝ | ⃝ | ⃝ | ⃝ | ⃝ | ⃝ |
| Social media (e.g., Facebook, Instagram, WhatsApp, Twitter, WeChat, Weibo, Line, QQ, etc.) | ⃝ | ⃝ | ⃝ | ⃝ | ⃝ | ⃝ |
| TV | ⃝ | ⃝ | ⃝ | ⃝ | ⃝ | ⃝ |
| Newspapers or magazines | ⃝ | ⃝ | ⃝ | ⃝ | ⃝ | ⃝ |
| Bar / pub, nightclub, karaoke place or restaurant | ⃝ | ⃝ | ⃝ | ⃝ | ⃝ | ⃝ |

1. During the past 6 months, did you see advertising or promotions for waterpipe / shisha / hookah in the following channels in Hong Kong?

|  | Never | 1  time | 2  times | 3  times | 4 or more times | Don’t remember / don’t know |
| --- | --- | --- | --- | --- | --- | --- |
| Convenience store (e.g., 7-11, Circle K, Vingo, etc.), local store, market, supermarket or gas station | ⃝ | ⃝ | ⃝ | ⃝ | ⃝ | ⃝ |
| Internet (e.g., taobao, YouTube, etc.) | ⃝ | ⃝ | ⃝ | ⃝ | ⃝ | ⃝ |
| Social media (e.g., Facebook, Instagram, WhatsApp, Twitter, WeChat, Weibo, Line, QQ, etc.) | ⃝ | ⃝ | ⃝ | ⃝ | ⃝ | ⃝ |
| TV | ⃝ | ⃝ | ⃝ | ⃝ | ⃝ | ⃝ |
| Newspapers or magazines | ⃝ | ⃝ | ⃝ | ⃝ | ⃝ | ⃝ |
| Bar / pub, nightclub, karaoke place or restaurant | ⃝ | ⃝ | ⃝ | ⃝ | ⃝ | ⃝ |

1. Regulations on Tobacco Products
2. Regarding the regulations on electronic cigarettes in Hong Kong, do you agree or disagree with the following statements?

|  | Strongly agree | Agree | Neither agree or disagree | Disagree | Strongly disagree | Refuse to answer |
| --- | --- | --- | --- | --- | --- | --- |
| Electronic cigarettes should be prohibited to sale to minors aged below 18 | ⃝ | ⃝ | ⃝ | ⃝ | ⃝ | ⃝ |
| Hong Kong should prohibit the use of flavors in electronic cigarettes (e.g., fruit, candy, mint, etc.) | ⃝ | ⃝ | ⃝ | ⃝ | ⃝ | ⃝ |
| Electronic cigarette advertising should be totally prohibited in Hong Kong (e.g., Internet, social media, TV, radio, newspaper, magazine, etc.) | ⃝ | ⃝ | ⃝ | ⃝ | ⃝ | ⃝ |
| Electronic cigarettes should be completely banned in Hong Kong, including import and sale | ⃝ | ⃝ | ⃝ | ⃝ | ⃝ | ⃝ |

1. Regarding the regulations on waterpipe / shisha / hookah in Hong Kong, do you agree or disagree with the following statements?

|  | Strongly agree | Agree | Neither agree or disagree | Disagree | Strongly disagree | Refuse to answer |
| --- | --- | --- | --- | --- | --- | --- |
| Waterpipe / shisha / hookah should be prohibited to be serviced or sold to minors aged below 18 | ⃝ | ⃝ | ⃝ | ⃝ | ⃝ | ⃝ |
| Hong Kong should prohibit the use of flavors in waterpipe / shisha / hookah (e.g., fruit, candy, mint, etc.) | ⃝ | ⃝ | ⃝ | ⃝ | ⃝ | ⃝ |
| Waterpipe / shisha / hookah advertising should be totally banned (e.g., Internet, social media, TV, radio, newspaper, magazine, etc.) | ⃝ | ⃝ | ⃝ | ⃝ | ⃝ | ⃝ |
| Waterpipe / shisha / hookah should be completely banned in Hong Kong, including import, sale and service in waterpipe lounges | ⃝ | ⃝ | ⃝ | ⃝ | ⃝ | ⃝ |

1. Email address used as coupon redemption

| Thank you for your participation! The first 200 participants who completed and submitted the questionnaire will receive a HK$50 Starbucks e-giftcard as a token of appreciation. Please kindly provide your email address. We will send you the e-giftcard by email if your submission is verified to be the first 200 valid questionnaires:  □ My email address is: ______________________  □ I do not need a HK$50 Starbucks e-giftcard.  Your personal information will be kept confidential and be destroyed within 6 months after the survey is complete. |
| --- |

| Submit questionnaire |
| --- |

Questionnaire completed

| The questionnaire is successfully submitted, thank you for your participation! |
| --- |
